# Anti-aphrodisiac pheromone, a renewable signal in adult butterflies

**DOI:** 10.1038/s41598-019-50838-1

**Published:** 2019-10-03

**Authors:** Raimondas Mozuraitis, Rushana Murtazina, Javier Zurita, Yuxin Pei, Leopold Ilag, Christer Wiklund, Anna Karin Borg Karlson

**Affiliations:** 1 Department of Zoology, Stockholm University, SE-10691 Stockholm, Sweden; 2 Laboratory of Chemical and Behavioural Ecology, Institute of Ecology, Nature Research Centre, Akademijos 2, LT-08412 Vilnius, Lithuania; 3 Department of Chemistry, School of Engineering Sciences in Chemistry, Biotechnology and Health, Royal Institute of Technology, KTH, SE-100 44 Stockholm, Sweden; 4 Department of Environmental Science and Analytical Chemistry, Stockholm University, SE-10691 Stockholm, Sweden; 5 Northwest A&F University, Department of Applied Chemistry, Yangling, Shaanxi 712100 People’s Republic of China; 6 Division of Organic Chemistry, Institute of Technology, Tartu University, Tartu, 50411 Estonia

**Keywords:** Behavioural ecology, Entomology

## Abstract

The male butterfly *Pieris napi* produces the anti-aphrodisiac pheromone methyl salicylate (MeS) and transfers it to the female during mating. After mating she releases MeS, when courted by conspecific males, which decreases her attractiveness and the duration of male harassment, thus increasing her time available for egg-laying. In previous studies we have shown that males produced MeS from the amino acid L-phenylalanine (L-Phe) acquired during larval stage. In this study we show that adult males of *P. napi* can utilize L-Phe and aromatic flower volatiles as building blocks for production of anti-aphrodisiac pheromone and transfer it to females during mating. We demonstrate this by feeding butterflies with stable isotope labelled molecules mixed in sugar solutions, and, to mimic the natural conditions, we fed male butterflies with floral nectar of *Bunias orientalis* plants treated with labelled L-Phe. The volatiles from butterflies and plants were collected and identified by solid phase micro extraction, gas chromatography and mass spectrometry techniques. Since *P. napi* is polygamous, males would gain from restoring the titre of MeS after mating and the use of aromatic precursors for production of MeS could be considered as an advantageous trait which could enable butterflies to relocate L-Phe for other needs.

## Introduction

After mating, female butterflies become unreceptive for either the rest of their lives in monandrous species or for a certain period in polyandrous species^1,2^. Moreover, male butterflies are incapable of forcing mating on females, so female receptivity is a prerequisite to successful male courtship^2^. Consequently, males gain little from courting unreceptive females. In a number of butterfly species, mated females signal unreceptiveness by releasing an anti-aphrodisiac, a pheromone transferred from males to females during mating that reduce attractiveness of females to subsequent courting males^3–6^. Therefore, anti-aphrodisiac pheromone represents an honest signal of female mating status, as both sexes benefit from not wasting time and energy through male harassment of unreceptive females^3,4^. In polygamous species, males are capable of mating several times. To maximize the duration of time that a female remains unreceptive, males transfer a large spermatophore during the first mating^7^, which in *Pieris napi* L (Lepidoptera: Pieridae) males corresponds to over 20% of their body mass^8^. Previous experiments have shown that the amount of anti-aphrodisiac released by *P. napi* females, having received a male’s first ejaculate, was on average 2.5 times higher than that released by females that had received a male’s second ejaculate, when the male re-mated on the day following his first mating^9^. This reflects the observation that male *P. napi* that mate so soon after their first mating transfer a spermatophore that is 2–3 times smaller than that transferred at their first mating^10,11^. Surprisingly, female *P. napi* mate as willingly with recently mated males as with virgin males, although they pay a double cost in terms of receiving a smaller spermatophore and suffer a longer time spent in copula^12^. When the stores of the anti-aphrodisiac are depleted, it stands to reason that males would benefit from being able to restore the amounts of anti-aphrodisiac during the adult stage after each mating.

However, anti-aphrodisiac also has trade-offs, such as their use as kairomones by natural enemies. Studies have shown that egg parasitoid wasps of two closely related *Trichogramma* species detect the anti-aphrodisiac odour released by mated *Pieris brassicae* and *Pieris rapae* females. The wasps then ride on butterfly females to egg-laying sites and then parasitize the freshly laid eggs^13,14^. Moreover, it was shown that risk of egg parasitoid attraction depends on anti-aphrodisiac titre in *P. brassicae* females^15^. Hitchhiking egg parasitoid wasps of *Trichogramma* species were detected on mated *P. napi* females as well^16^, demonstrating that those wasps exploit anti-aphrodisiacs of all three *Pieris* species. When parasitoid density is high, this could cause a selective pressure on amounts of aphrodisiac used for pheromone communication.

As a model to test our hypothesis that adult males can produce and transfer the anti-aphrodisiac to the females we have selected polygamous butterflies of *P. napi*, which use methyl salicylate (MeS) as anti-aphrodisiac pheromone^3^. MeS is a ubiquitous multifunctional molecule that is naturally released from leaves and flowers of numerous plant species^17^. It is emitted by conifers infested by arthropods^18^; it attracts or repels herbivores depending on the identity of the plant-herbivore system like in aphids where it acts as a repellent from the winter host^19^; it functions as an antifeedant for pine weevils^20^, and it reduces egg-laying by the cabbage moth^21^. Additionally, MeS also functions as an aggregation-attachment pheromone of the tropical bont tick *Amblyomma variegatum*^22^, and lures natural enemies of the herbivores towards the damaged site of a plant^23^. The compound also has the ability to attract pollinators to flowers^24^. Finally, MeS has been identified in mandibular gland secretions of three ant species of the genus *Myrmecocystus*. The compound caused excited movements in the *M. mexicanus* ant species functioning as a component of alarm pheromone^25^.

Previous studies have shown that males produce anti-aphrodisiac from precursors acquired during larval feeding^3–5^, however there are no studies reporting male butterflies’ ability to produce anti-aphrodisiac from resources acquired during adult feeding.

Our previous experiments showed that for the ant-aphrodisiac production, males of three *Pieris* species use L-phenylalanine (L-Phe) as a precursor to MeS and benzyl cyanide as well as L- tryptophan for production of indole^3,4^. To the best of our knowledge, there are no studies dealing with biosynthesis of MeS in animals. In plants and microorganisms MeS is derived from the shikimate pathway in two different ways, either via L-Phe or via isochorismic acid^26,27^. Biosynthesis of MeS from L-Phe is carried out via a number of aromatic intermediates (Supplementary Fig. 1), some of which are also released by flowers of plant species to attract pollinators^28^ including *Pieris* butterflies. These aromatic compounds can partly dissolve in nectar and could, together with L-Phe, serve as potential precursors for production of MeS in adult males.

In this study our goals were i) to show that adult butterfly males are able to biosynthesize their anti-aphrodisiac pheromone from their intake of labelled L-Phe and from synthesized labelled aromatic substances identified in the scent and the nectar of *Bunias orientalis* L (Brassicales: Brassicaceae) flowers and; ii) to follow the transfer of the anti-aphrodisiac from male to female during mating and emission of the pheromone during her mate-refusal posture.

## Materials and Methods

**Figure 1:**
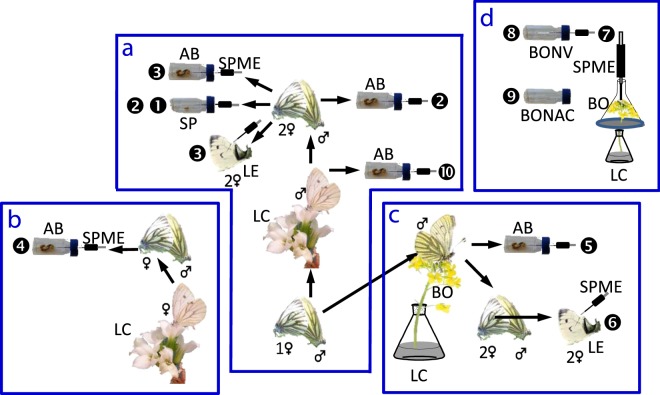
Conceptual figure summarising the study design. The figure shows the experiments (**a**) fulfilling aims 1–3 and 10; (**b**) revealing whether adult females are able to produce the anti-aphrodisiac pheromone; (**c**) revealing whether adult butterflies are able to utilise the naturally occurring precursor(s) for anti-aphrodisiac production; (**d**) used to identify volatiles and amino acids in floral nectar of *Bunias orientalis* plants. Number in a circle indicates an aim; ➊ to reveal whether adult males are able to produce the anti-aphrodisiac pheromone from their intake of synthetic labelled L-Phenylalanine (L-Phe) in the adult stage and to transfer it into spermatophore during mating; ➋ to determine whether the ratio of labelled and non-labelled molecular ions is the same in the volatiles sampled from spermatophore and crushed male abdomen; ➌ to reveal whether the mated females emit the labelled volatiles received in a spermatophore from the males and to compare the ratio of labelled and non-labelled molecular ions in the volatiles released by live female and by crushed abdomen of the female; ➍ to show whether the adult females are able to produce the anti-aphrodisiac; ➎ reveal whether adult males are able to produce the anti-aphrodisiac from intake of naturally occurring precursor(s) by feeding on a nectar of the plants frequently visited by butterflies under natural conditions; ➏ to show whether the mated females emit the labelled volatiles received from the males fed on flowers that have been immersed in a solution with labelled L-Phe; ➐ to reveal whether the aromatic floral volatiles of *B. orientalis* plants immersed in a solution with labelled L-Phe contain labelled moiety and to determine the level of incorporation; ➑ to determine which aromatic floral volatiles are released from nectar of *B. orientalis* flowers; ➒ to determine which amino acids are present in nectar of *B. orientalis* flowers; ➓ to test whether adult males are able to produce the anti-aphrodisiac pheromone from their intake of synthetic labelled aromatic compounds analogous to the aromatic volatiles produced by *B. orientalis* flowers. The labels indicate: AB = abdomen; SP = spermatophore; LE = life emission; SPME = solid phase micro extraction; LC = labelled compounds; BO = *B. orientalis* flowers; BONV = *B. orientalis* nectar volatiles; BONAC = *B. orientalis* nectar amino acids; a number before a female symbol indicates identity of females used in stages of the experiment.

**Figure 2:**
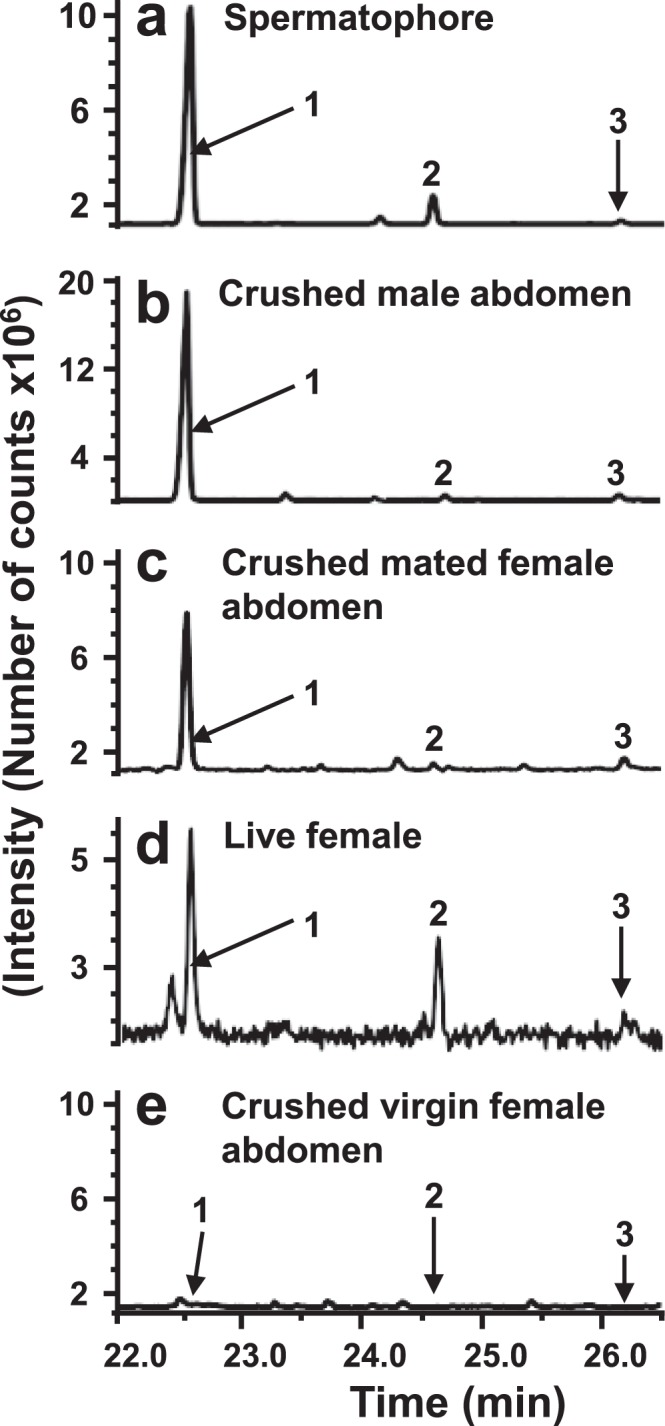
Representative total ion chromatograms of volatiles sampled from *Pieris napi* (**a**) spermatophore, (**b**) crushed male abdomen, (**c**) crushed mated female abdomen, (**d**) live female showing mate-refusal posture, and (**e**) crushed virgin female abdomen. Peaks are indicated as follows: (1) methyl salicylate, (2) 2-methoxyphenol, and (3) benzyl cyanide. The compound eluting before methyl salicylate in live female emission is an impurity from the SPME absorbent. The following amounts of methyl salicylate (40–60 ng), 2-methoxyphenol (6–8 ng), and benzyl cyanide (2–3 ng) were collected on SPME fibre from the headspace above spermatophore.

To fulfil these goals, we set up ten aims (Fig. 1). The **first aim** was formulated to reveal whether adult males are able to produce the anti-aphrodisiac pheromone from their intake of synthetic labelled L-Phe at the adult stage and to transfer it into the spermatophore during mating. To show this, we used non-virgin, males, i.e. males that had already delivered a first supply of anti-aphrodisiac pheromone produced from L-Phe ingested at the larval stage, so as to increase the possibility to detect *de novo* biosynthesis of the anti-aphrodisiac in the adult butterfly males. Hence, the first-time-mated males were allowed to mate a second time, after feeding on stable isotope ^13^C_6_–labelled-L-Phe (Larodan Fine Chemicals, Malmö, Sweden) (nine males) and 2,6-D_2_-benzyl alcohol (seven males) for two days. The undamaged spermatophores from six and three females mated with labelled L-Phe and 2,6-D_2_-benzyl alcohol fed males, respectively, were dissected out of the abdomens, individually transferred to sampling vials, crushed and used for volatile collection and analysis (Fig. 2, Table 1).

**Table 1:** Ratio of labelled and non-labelled molecular ions in the compounds present in effluvia of male *Pieris napi* spermatophore and crushed abdomen after feeding on labelled L-phenylalanine and benzyl alcohol.

Compounds in spermatophore	Males fed L-phenylalanine		Males fed benzyl alcohol	
	Spermatophore	Abdomen	Spermatophore	Abdomen
Methyl salicylate	0.4 ± 0.08**	0.4 ± 0.06***	0.7 ± 0.13*	0.7 ± 0.10**
2-Methoxyphenol	0.2 ± 0.07**	0.2 ± 0.05***	0.4 ± 0.11*	0.4 ± 0.09**
Benzyl cyanide	0.6 ± 0.11**	0.6 ± 0.09**	nl, *P* = 0.83	nl, *P* = 0.75

Numbers are means followed by standard error; nl indicates not labelled as the nitrogen containing benzylcyanide cannot be formed from benzylalcohol; number of replicates used for: L-phenylalanine spermatophore n = 6, L-phenylalanine abdomen n = 9, benzyl alcohol spermatophore n = 3, benzyl alcohol abdomen n = 7; significance of incorporation of the labelled moieties into the compounds was determined by Mann-Whitney U-Tests, (*P* values: *** < 0.001, ** < 0.01, * < 0.05). The following amounts (mean ± SEM) of methyl salicylate (126 ± 24 ng), 2-methoxyphenol (20 ± 7 ng), and benzyl cyanide (2 ± 1 ng) were collected on SPME fibre from the headspace above spermatophore. The spermatophores have been dissected from the females mated with the males fed on L-phenylalanine.

Dissection of a spermatophore from an abdomen of a female has to be done immediately after mating, which is time consuming and requires accuracy. To simplify the experimental procedure, we formulated the **second aim** to test whether the ratio of labelled and non-labelled molecular ions is the same in the volatiles sampled from spermatophore and crushed male abdomen. To fulfil the aim, the males used in the previous experiment were immediately frozen after their second mating and stored at −18 °C a few days until analysis. The ratios of labelled and non-labelled molecular ions and volatile profiles from the spermatophores and crushed abdomens were compared (Fig. 2, Table 1).

For the **third aim**, we aimed to reveal whether mated females emit the labelled volatiles received in a spermatophore from males and to compare the ratio of labelled and non-labelled molecular ions in the volatiles released by live female and by crushed abdomen of the female. In this experiment five, four and four virgin females were mated with non-virgin males, fed on ring ^13^C_6_–labelled-L-Phe, 2,6-D_2_-benzyl alcohol or 2,6-D_2_-benzoic acid respectively, after their first mating (Table 2). The day after mating, sampling of volatiles released from the females displaying mate refusal posture was carried out for 1 hour. Afterwards, females were immediately frozen and stored at −18 °C for a few days until sampling of volatiles from their crushed abdomens was carried out. In addition to the females used in the emission sampling experiments, another three and one females, mated with males fed on ^13^C_6_–labelled-L-Phe and 2,6-D_2_-benzyl alcohol respectively, were frozen after mating for determination of incorporation ratio of labelled moiety in volatiles sampled from crushed abdomens.

**Table 2:** Ratio of the intensity of labelled and non-labelled ions in the aromatic compounds present in effluvia of female *Pieris napi* abdomen and in emission of live females, mated with males fed on labelled substrates.

*P. napi* biosynthesized compounds	Females mated with males fed labelled					Females fed labelled L-Phe, mated with males not fed labelled L-Phe
	L-Phenylalanine		Benzoic acid		Benzyl alcohol	
	Abdomen	Live	Abdomen	Live	Live	Abdomen
Methyl salicylate	0.9 ± 0.6***	0.9 ± 0.2**	0.4 ± 0.02**	0.4 ± 0.07**	0.9 ± 0.1**	nl, *P* = 0.83
2-Methoxyphenol	0.5 ± 0.4**	pi, *P* = 0.14	0.03 ± 0.04*	nl, *P* = 0.68	pi; *P* = 0.17	nl, *P* = 0.78
Benzyl cyanide	0.9 ± 0.5***	pi, *P* = 0.12	nl, *P* = 0.78	nl, *P* = 0.84	nl, *P* = 0.72	nl, *P* = 0.59

Numbers are means followed by standard error; Live are emission of live females; nl indicates not labelled, pi indicates possible incorporation; L-Phe indicates L-phenylalanine; number of replicates used for: L-phenylalanine abdomen N = 8, L-phenylalanine live N = 5, Benzoic acid abdomen N = 5, Benzoic acid live N = 4, Benzyl alcohol live N = 4, Females fed labelled L-Phe, mated with males not fed labelled L-Phe N = 3; significance of incorporation of the labelled moieties into the compounds was determined by Mann-Whitney U-Tests, (*P* values: *** < 0.001, ** < 0.01, * < 0.05).

The **fourth aim** was set up to show whether the adult females are able to produce anti-aphrodisiac. Three adult females were fed on ring ^13^C_6_–labelled-L-Phe for four days and then mated with the first-time-mated males fed non-labelled-L-Phe. Volatiles from the crushed abdomens of these females were sampled and analysed (Table 2).

The **fifth aim** was set up to reveal whether adult males are able to produce the anti-aphrodisiac from an intake of naturally occurring precursor(s) by feeding them on a nectar of the plants frequently visited by butterflies under natural conditions. *Bunias orientalis* L (*Brassicales: Brassicaceae*) plants were selected as a model species as flowers of those plants did not release any of the three compounds produced and transferred by *P. napi* males to the female at mating. We cut stems of *B. orientalis* with buds and newly opened flowers and placed them in 0.1% of labelled L-Phe (99% ring- D_5_–labelled, Cambridge Isotope Laboratories, Tewksbury, MA, USA) water solution for three days. Afterwards, we allowed the first-time-mated males to feed on the flowers which were immersed in labelled L-Phe water solution and after two days feeding, six males were frozen and stored at −18 °C for a few days until analysis (Table 3).

**Table 3:** Ratio of labelled and non-labelled molecular ions in flower and nectar volatiles of *Bunias orientalis* immersed in solution with labelled L-phenylalanine as well as in effluvia of *Pieris napi* male abdomen after feeding on those flowers and emission of live females mated with males after feeding on those flowers.

Compounds	Flower fragrance	Nectar	Male abdomen	Female emissions
Phenylacetaldehyde	10.5 ± 1.0**	x	nd	nd
2-Phenylethanol	11.9 ± 0.3**	x	nd	nd
Benzyl acetate	9.6 ± 0.1**	nd	nd	nd
Benzaldehyde	4 ± 0.6**	x	nd	nd
Benzyl alcohol	41.3 ± 34**	x	nd	nd
Benzoic acid	nd	x	nd	nd
Methyl benzoate	3.2 ± 3.2**	nd	nd	nd
Cinnamaldehyde	0.5 ± 0.3**	nd	nd	nd
Cinnamyl alcohol	1.8 ± 1.4**	x	nd	nd
Methyl salicylate	nd	nd	0.03 ± 0.009*	0.03 ± 0.013*
2-Methoxyphenol	nd	nd	0.02 ± 0.008*	0.01 ± 0.007*
Benzyl cyanide	nd	nd	0.01 ± 0.005*	nl, *P* = 0.65

Numbers are means followed by standard errors of means; nd indicates that compound was not detected; x indicates that compound was present in nectar; nl indicates not labelled; Male abdomen indicates crushed male abdomen, Female emissions indicates emission from female showing refusal posture; *Bunias orientalis* flower fragrance N = 5, *Bunias orientalis* nectar N = 2, Male abdomen N = 6, Female emissions N = 4; significance of incorporation of the labelled moieties into the compounds was determined by Mann-Whitney U-Tests, (*P* values: *** < 0.001, ** < 0.01, * < 0.05).

For the **sixth aim**, we aimed to show whether the mated females emit the labelled volatiles received from the males fed on flowers that have been immersed in solution with labelled L-Phe. We used another four males treated in the same way as described in the experiment of the objective five, but instead of freezing the males after feeding, we allow them to mate with virgin females and the day after mating, we sampled the volatiles released from the females displaying mate refusal posture.

In plants, biosynthesis of MeS from Phe is carried out via number of aromatic intermediates some of which are released by flowers and could serve as potential precursors for production of MeS in adult males. Hence, the **seventh aim** was set up to reveal whether the aromatic floral volatiles of *B. orientalis* plants immersed in solution with labelled L-Phe contain labelled moiety and to determine the level of incorporation. To show this, we individually sampled floral volatiles from five cut inflorescences immersed in 0.1% of non-labelled L-Phe solution in water for three days, as well as from another five inflorescences immersed in 0.1% of labelled L-Phe solution in water. The control group of the plants was used to calculate the level of incorporation of labelled L-Phe moiety into aromatic floral volatiles (Table 3).

The **eighth aim** was set up to determine which aromatic floral volatiles are released from nectar of *B. orientalis* flowers (Table 3) and the **ninth aim** served to determine which amino acids are present in nectar of *B. orientalis* flowers (Table 3, Fig. 4).

The **tenth aim** was formulated to test whether adult males are able to produce the anti-aphrodisiac pheromone from their intake of synthetic labelled aromatic compounds analogous to the aromatic volatiles produced by *B. orientalis* flowers and present in the nectar. To show this, the first-time-mated males were fed on the compounds labelled with deuterium on positions 2 and 6, namely 2,6- D_2_-benzaldehyde (five males), 2,6- D_2_-benzyl alcohol (seven males), 2,6- D_2_-benzoic acid (six males), 2,6- D_2_-methyl benzoate (four males), 2,6- D_2_-benzyl cyanide (four males), 2,6-D_2_-methyl cinnamate (five males), and 2-phenylethanol (six males). The labelled substrates were offered together with sucrose on odourless *K. blossfeldiana* flowers during 2 days (Table 4). For detailed descriptions of the synthesis of these labelled substrates, see Supplementary Information. After feeding, males were frozen and stored at −18 °C for a few days until sampling of volatiles from crushed abdomens.

**Table 4:** Ratio of labelled and non-labelled ions of aromatic compounds in effluvia of *Pieris napi* male abdomen after feeding on labelled substrates.

Male biosynthesized compound	Male feeding substrate							
	Benz aldehyde	Benzyl alcohol	Benzoic acid	Methyl benzoate	Benzyl cyanide	Methyl cinnamate	2-Phenyl ethanol	L-Phenyl alanine
Methyl salicylate	0.4 ± 0.08**	0.7 ± 0.1**	0.4 ± 0.09**	0.2 ± 0.03**	0.2 ± 0.07**	0.4 ± 0.04**	nl, *P* = 0.87	0.4 ± 0.06***
2-Methoxyphenol	0.4 ± 0.06**	0.4 ± 0.09**	0.4 ± 0.07**	0.1 ± 0.03**	0.1 ± 0.04**	0.4 ± 0.08**	nl, *P = *0.63	0.2 ± 0.05***
Benzyl cyanide	nl, *P* = 0.92	nl, *P* = 0.57	nl, *P* = 0.75	nl, *P* = 0.39	not relevant	nl, *P* = 0.93	nl, *P* = 0.52	0.6 ± 0.1***

Numbers are means followed by standard error; not relevant indicates not measured due to possible contamination; nl indicates not labelled; number of replicates used for: benzaldehyde N = 5, benzyl alcohol N = 7, benzoic acid N = 6, methyl benzoate N = 4, benzyl cyanide N = 4, methyl cinnamate N = 5, 2-phenylethanol N = 6, L-phenylalanine N = 9; significance of incorporation of the labelled moieties into the compounds was determined by Mann-Whitney U-Tests, (*P* values: *** < 0.001, ** < 0.01, * < 0.05).

### Study species

*P. napi* is a widespread polygamous butterfly species in Europe, Asia, and North America. At the Department of Zoology, Stockholm University, the butterflies of this species are used as a model object in various studies, including our previous research dealing with anti-aphrodisiac pheromone. To avoid inbreeding, every year a few hundred eggs are collected from the natural habitat of the species and hatched larvae are bred under short day (8 hours light, 16 hours dark) laboratory conditions, directing pupae into diapause. The next year a few generations are produced for experimental needs. The founders of the laboratory population used in this study were collected near Stockholm University campus (59.36693°, 18.06848°), Stockholm, Sweden. The larvae were fed on *Alliaria petiolata* Scop (Brassicales: Brassicaceae) in laboratory conditions at a 22:2 h L:D photoperiod and 25 °C. The larval development time was 14 ± 2 days. Adult butterflies were fed with sucrose applied on the flowers of *Kalanchoe blossfeldiana* Poelln. (Saxifragales: Crassulaceae). This flowering plant was selected as it produced neither nectar nor odour, and consequently did not interfere with our chemical analyses.

*B. orientalis* is a widespread invasive plant in Sweden which is frequently visited by adult *P. napi* butterflies for feeding on nectar. Despite the nectar production per flower in *B orientalis* being relatively low^29^, the extremely high number of flowers per plant makes *B orientalis* valuable food source for pollinators including butterflies^30^. It is a common pattern that nectar-feeding butterflies visit various flowering plants and often feed at flowers with minute volumes of nectar^31^. The plants of this species were chosen for the study as their flowers neither produce MeS, benzyl cyanide nor methoxyphenol.

Inflorescences of *B. orientalis* were collected in mornings in June near the campus of the Royal Institute of Technology (KTH), Stockholm, Sweden (59.34911, 18.075385).

### Collection of butterfly odours

Butterfly odours were sampled from a headspace by solid phase micro extraction technique (SPME). For each individual, before sampling, the abdomen was detached from the rest of the body and crushed in a separate 1.7 ml vial, which was immediately closed by aluminium foil. A purified SPME fibre (65 µm polydimethylsiloxane/divinylbenzene, Supelco, USA) was inserted and the volatiles released were collected during 30 min. Afterwards these were analysed by gas chromatography and mass spectrometry techniques (GC-MS).

To collect odours emitted by live females, a glass cylinder (height 18 cm, diameter 8 cm, volume 90 cm^3^) was used with one small opening for inserting an SPME fibre^3^. Two females were placed together in the glass cylinder and when one female was flying the other female adopted the mate-refusal posture and thereby releasing volatiles in response to the flying butterfly. Sampling of volatiles lasted for 1 hour.

### Feeding butterflies with labelled substrates

All isotopically labelled compounds, except -L-Phe, were initially dissolved in ethanol and were added before feeding experiments to produce ~2% concentration by weight to a ~20% sucrose solution in water. Labelled L-Phe was dissolved directly in ~20% sucrose solution in water. The solutions were applied to the odourless flowers of *K. blossfeldiana* and butterflies were allowed to feed for two days.

### Sampling of floral volatiles from inflorescences and nectar

To identify the flower scent constituents, the volatiles from five *B. orientalis* inflorescences were collected by SPME. The flowers were placed in Toppits oven proof plastic bag (Cofresco®, Minden, Germany) and the SPME fibre inserted close to the inflorescence during one hour.

To identify what floral volatiles male butterflies were able to imbibe when feeding on flowers of *B. orientalis*, nectar of 50 flowers were collected using 1 microliter microcaps (Drummond Scientific Company, Broomall, PA, USA) and transferred to a 0.7 ml glass vial closed with autosampler screw cap bearing silikon/PTFE septum (Chromacol, Scantec Nordic, Göteborg, Sweden). The SPME fibre was inserted in the glass vial with nectar close to, but not touching, the nectar surface. Sampling time was 1 hour.

### Profiling amino acids in flower nectar

The amino acid profiles in nectar of *B. orientalis*, was identified by derivatization of the free amino acids with *N*-hydroxysuccinimide ester of N-butylnicotinic acid (C_4_-NA-NHS)^32^, followed by identification using liquid chromatography tandem mass spectrometry (LC-MS/MS) in multiple reaction monitoring (MRM) mode (for more details, see Supplementary Information).

### Separation and identification of volatiles

Separation and identification of the flower and butterfly volatiles were made by GC-MS using a Varian 3400 gas chromatograph (GC) (Varian, Palo Alto, CA, USA) coupled with a Finnigan SSQ 7000 mass spectrometer (MS) (Thermo-Finnigan, San Jose, CA, USA). A DB-WAX column (length 30 m, inner diameter 0.25 mm, and film thickness 0.25 μm; J & W Scientific, Folsom, CA, USA). The temperature programme started isothermal at 40 °C for 1 min, then was increased 5 °C/min to 220 °C and afterwards was held isothermal for 10 min. The injector temperature was kept isothermal at 225 °C and helium was used as the carrier gas at 69 kPa. The mass spectrometer was operated with full scan mode (mass range m/z 30–400 m/z) and mass spectra were obtained at 70 eV with the ion source at 150 °C. Identification of the compounds was made by comparison of their retention times and mass spectra with those of authentic reference standards as well as by comparing mass spectral data of natural products with those available from NIST mass spectral data base, version 2.0 (National Institute of Standards and Technology, USA).

### Determination of the ratio of isotope incorporation

The level of incorporation in feeding experiments was determined as the ratio between the abundance of labelled molecular ion ($${M}_{+n})$$ and the non-labelled molecular ion ($${M}_{+})$$ in compounds of treated samples minus the ratio of the abundance of the natural isotope ions ($${M}_{+n})$$ and the molecular ions ($${M}_{+})$$ of compounds in control (non-labelled) samples.

$${\rm{Ratio}}\,{\rm{of}}\,{\rm{incorporation}}={({{\rm{M}}}_{+{\rm{n}}}/{{\rm{M}}}_{+})}_{{\rm{treated}}}-{({{\rm{M}}}_{+{\rm{n}}}/{{\rm{M}}}_{+})}_{{\rm{control}}}$$

where n = 1 for methyl salicylate and 2-metoxyphenol; n = 2 for benzyl cyanide, after feeding on 2,6-D_2_-benzyl alcohol, 2,6-D_2_-benzaldehyde, 2,6- D_2_-methyl benzoate, 2,6-D_2_-benzoic acid, 2,6-D_2_-benzyl cyanide, 2,6-D_2_-methyl cinnamate; n = 4 for methyl salicylate and 2-metoxyphenol; n = 5 for methyl salicylate, 2-metoxyphenol and benzyl cyanide after feeding on flowers of *B. orientalis* that were immersed in ring D_5_-L-Phe water solution; and n = 6 for methyl salicylate, 2-metoxyphenol and benzyl cyanide after feeding mated males, virgin females and mated females with ^13^C_6_-L-Phe.

The control (non-labelled) MeS, 2-metoxyphenol and benzyl cyanide were sampled from crushed abdomens of nine males, which after the first-time-mating were allowed to feed on non–labelled-L-Phe for two days.

### Statistical analyses

All statistical analyses were conducted with Statistica software version 9 (StatSoft, US). We used nonparametric Mann-Whitney U-test to determine whether incorporation of the labelled moieties into the compounds was significant by comparing the ratio between the labelled molecular ion ($${M}_{+n})$$ and the non-labelled molecular ion ($${M}_{+})$$ in compounds of treated samples versus the ratio of the labelled molecular ions ($${M}_{+n})$$ and the molecular ions ($${M}_{+})$$ of compounds in control (non-labelled) samples. Control samples were obtained from the crushed abdomens of males, which after the first mating were fed on non- labelled L-phenylalanine for two days. We used Wilcoxon’s matched pairs test to compare between the ratios of incorporation in the target compounds released from crushed abdomen versus spermatophore.

## Results

### Production of anti-aphrodisiac pheromone by adult males and transfer of the pheromone to females

**Figure 3:**
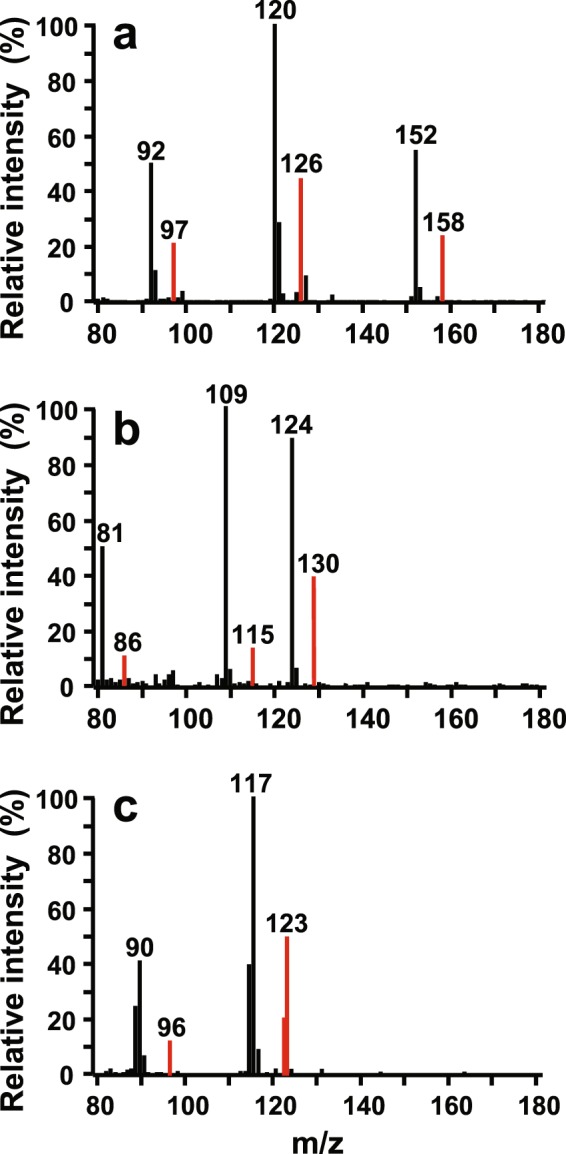
Mass spectra of (**a**) methyl salicylate, (**b**) 2-methoxyphenol, and (**c**) benzyl cyanide from abdomen of *Pieris napi* male fed on ^13^C_6_-labelled L-phenylalanine. M_n_ = 152 and M_n+6_ = 158 are molecular ions of non-labelled and labelled methyl salicylate, respectively; M_n_ = 124 and M_n+6 = _130 are molecular ions of non-labelled and labelled 2-methoxyphenol, respectively; M_n_ = 117 and M_n+6_ = 123 are molecular ions of non-labelled and labelled benzyl cyanide, respectively; most abundant ions originating from ^13^C_6_-labelled L-phenylalanine are indicated by red colour.

Adult male butterflies fed on ring ^13^C_6_ labelled L-phenylalanine (L-Phe) showed incorporation of the ^13^C labelled aromatic ring in methyl salicylate (MeS), anti-aphrodisiac pheromone. Hence, the males were able to increase the anti-aphrodisiac amounts by at least 40% compared to the anti-aphrodisiac level the males had after the first mating (Table 1). In addition to MeS, incorporation was registered in 2-methoxyphenol and benzyl cyanide (Figs 2a and 3a–c, Table 1). The ratio of incorporation of ^13^C atoms in MeS was significantly higher than in 2- methoxyphenol but lower than in benzyl cyanide (Mann-Whitney U-Tests, *P* = 0.04 and *P* = 0.03, respectively) (Table 1).

The comparison of emissions from spermatophores between those from crushed male and mated female abdomens revealed that MeS, 2-methoxyphenol and benzyl cyanide released from spermatophores were also present in the emissions from crushed abdomens of males and mated females (Fig. 2a–c, Table 1). Incorporation of the ^13^C labelled aromatic ring in MeS, 2-methoxyphenol and benzyl cyanide released from spermatophores were similar to those released from crushed male abdomens (Wilcoxon’s matched pairs test, *P* = 0.93, *P* = 0.84 and *P* = 0.97, respectively). Moreover, the spermatophore and crushed abdomens of males fed on deuterium labelled benzylalcohol contained deuterium labelled MeS and 2-methoxyphenol with similar incorporation rates (Wilcoxon’s matched pairs test, *P* = 0.74 and *P* = 0.59, respectively) while benzyl cyanide was not labelled (Table 1). Hence, analyses of the crushed male abdomens reflected the incorporation ratio of labelled moieties and content of the volatiles released from the spermatophores which are transferred to the females during mating. Consequently this method allows determination of incorporation ratio without taking spermatophores out of the abdomens.

In females, mated with males fed on labelled L-Phe emitted labelled MeS, and possibly labelled benzyl cyanide and 2-methoxyphenol (Fig. 2d, Table 2), volatiles from crushed abdomens of the females contained labelled MeS, 2-methoxyphenol and benzyl cyanide. Methyl salicylate was the only significantly labelled compound released from females after mating with males fed on deuterium labelled benzoic acid and benzyl alcohol, whereas benzyl cyanide was not labelled (Table 2).

Analysis of compounds released from abdomens of *P. napi* females fed on labelled L-Phe and mated with non-labelled control males revealed three non-labelled aromatic compounds: MeS, 2-methoxyphenol and benzyl cyanide (Table 2). These compounds were not detected in the samples obtained from the crushed abdomens of virgin females (Fig. 2e).

### Floral aromatic volatiles from *B. orientalis* treated with labelled L-phenylalanine

*Bunias orientalis* inflorescenses with their stems immersed in 0.1% D_5_–labelled L-Phe emitted eight labelled compounds: phenylacetaldehyde, 2-phenylethanol, benzyl acetate, benzaldehyde, benzyl alcohol, methyl benzoate, cinnamaldehyde and cinnamyl alcohol. The incorporations, measured by isotopic ratio of the molecular ions, were different for each floral constituent (Table 3).

### Amino acids and aromatic volatiles in nectar of *B. orientalis*

**Figure 4:**
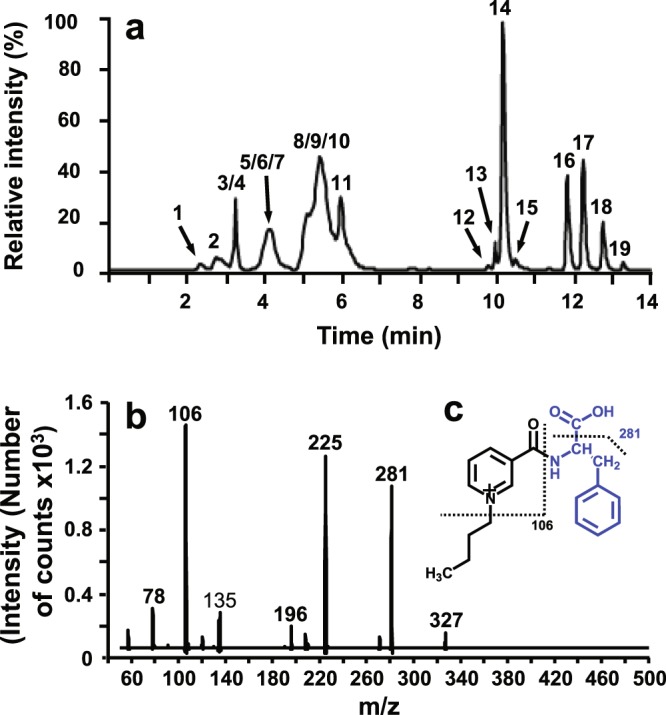
(**a**) Multiple reaction monitoring (MRM) chromatogram of *Bunias orientalis* nectar, 1: histidine, 2: asparagine, 3: arginine, 4: serine, 5: glycine, 6: glutamine, 7: aspartic acid, 8: glutamic acid, 9: threonine, 10: proline, 11: alanine, 12: lysine, 13: tyrosine, 14: valine, 15: methionine, 16: isoleucine, 17: leucine, 18: phenylalanine, 19: tryptophan. (**b**) ion spectrum of phenylalanine, where the diagnostic transition 327 > 281 was monitored together with the non-diagnostic transition 327 > 106. This pattern of fragmentation was observed in almost all the other derivatized amino acids. (**c**) Production pathways of monitored ions.

The presence of amino acids in *B. orientalis* was confirmed by derivatization and LC-MSMS analysis. At least 19 amino acids were detected in the nectar, including two aromatic amino acids Phe and tryptophan (Fig. 4).

In the volatiles of *B. orientalis* nectar, five aromatic compounds including phenylacetaldehyde, 2-phenylethanol, benzaldehyde, benzylalcohol and cinnamylalcohol were identical to those present in the flower scent, while benzoic acid was only released from the nectar sample (Table 3).

### Production and transfer of anti-aphrodisiac by adult males to females after feeding on *Bunias* nectar treated with labelled L-phenylalanine

To mimic natural conditions for nectar feeding butterflies and visualize the transfer of building blocks from flower to female, males were, after their first mating, allowed to feed on nectar from D_5_-L-Phe labelled host plants. Volatiles collected from the abdomen of these males revealed incorporation of labelled MeS, 2-methoxyphenol and benzyl cyanide (Table 3). Another group of males were, after their first mating, allowed to feed on nectar from D_5_-L-Phe labelled host plants and mate with virgin females. Volatiles collected from the mated female displaying refusal posture revealed labelled MeS and 2-methoxyphenol (Table 3)

### Utilization of synthetic flower scent constituents for production of anti-aphrodisiac by adult males

After showing that males incorporated substances from their nectar-intake into the biosynthesis of the anti-aphrodisiac, a new group of males were fed with deuterium labelled synthetic analogues of the compounds present in the flower volatiles that were also identified from the scent of flower nectar (Table 3, for synthesis and identity of the deuterium labelled compounds see Supplementary Information). Analyses showed that MeS and 2-methoxyphenol were biosynthesized from all labelled non-nitrogen containing substrates fed to the males, except for 2-phenylethanol which has a two carbon chain. Benzyl cyanide was labelled only when butterflies were fed on labelled L-Phe (Table 4).

## Discussion

In previous work we have shown that methyl salicylate (MeS) acts as an anti-aphrodisiac pheromone in two *Pieris* species^3,4^ and with ^13^C-labelling we demonstrated that only males of *P. napi* are able to produce MeS from labelled L-phenylalanine (L-Phe) acquired in the larval stage, which at the first mating is transferred to the female^3^.

Here we demonstrate that adult *P. napi* males are able to produce the anti-aphrodisiac pheromone MeS from L-Phe and also a number of aromatic volatiles commonly present in floral nectar; thus partly restoring the titer of anti-aphrodisiac needed for the next mating. By using sugar solution spiked with either ^13^C labelled L-Phe or a number of deuterium labelled aromatic substances we showed that the adult males were able to transfer the newly biosynthesized MeS from precursors originating from floral nectar to females at mating. In addition to MeS, benzyl cyanide and 2-methoxyphenol incorporated labelled atoms and were also transferred to the females during mating. These compounds have been detected in effluvia from females when adopting the mate-refusal posture, however, due to the low amounts of benzyl cyanide and 2-methoxyphenol trapped, the incorporation level was not significant. To the best of our knowledge, there are no data published showing a behaviour function of 2-methoxyphenol and benzyl cyanide for *P. napi* butterflies, while benzyl cyanide was identified as anti-aphrodisiac in the closely related species *Pieris brassicae*^4^. It is common knowledge that closely related species use the same biosynthetic route for their pheromones. We are planning to test whether those two compounds elicit olfactory responses and, if so, test their behavioural activity in *P. napi* species. Alternatively, 2-methoxyphenol and benzyl cyanide could be side products from the shared biosynthetic route. We predict that 2-methoxyphenol and benzyl cyanide are side products due to the low amounts produced, however, this prediction has yet to be tested.

To the best of our knowledge, there are no data published dealing with amino acid composition in nectar of *B. orientalis* flowers. In spite of nectar production per flower in *B orientalis* being relatively low^32^, our developed sensitive analytical method enabled us to detect 19 of 20 standard proteinogenic amino acids, including Phe that is used for production of anti-aphrodisiac by *P. napi* male amino acids, from around 1μl of nectar. Our data are in accordance with the results of a large scale screening of nectar samples by Baker and Baker^33^, revealing the presence of amino acids in 260 of 266 species tested and showing that amino acids are commonly present in floral nectars. Moreover, the authors compared the nectars from flowers pollinated by different pollinator guilds and concluded that nectars of butterfly visited flowers have higher concentrations of amino acids than those of flowers visited by other pollinators^33,34^. Later studies showed that all amino acids found in proteins, as well as non-protein amino acids, have been identified in various plant nectars^35–38^. In general Phe, serving as a precursor for anti-aphrodisiac production in *P. napi males*, is present at various ratios in nectar of a broad range of flowering plants^31,35,36,39,40^. In our study, the newly developed method was carried out to document its presence, rather than quantify Phe in the nectar of *B. orientalis* flowers. However, we may conclude that the amount of Phe that is present is apparently below average compared to those of other amino acids.

After treatment of *B. orientalis* plants with labelled L-Phe, the flowers emitted several labelled volatile aromatic compounds including benzaldehyde, benzylalcohol and methyl benzoate. These compounds were also found in the nectar together with benzoic acid and cinnamic alcohol, of which the latter substance is easily oxidized to cinnamic acid. The benzenoids are ubiquitous in floral volatiles of many plant species, including floral odours of *Pieris* host-plant species belonging to the family Brassicaceae^41,42^, and many of them serve as pollinator attractants^43,44^. The presence of volatiles in the nectar sample can be explained by their capacity to dissolve from adjacent floral tissues to the aqueous-sugar medium of nectar^45^. It has been demonstrated that several floral scent compounds released from flowers which do not produce nectar were taken up by artificial nectar added to the flowers^46^. In addition, microorganisms in nectar may degrade L-Phe and produce the volatiles identified.

We have shown that males of *P. napi* were able to use these volatile aromatic compounds for the production of the anti-aphrodisiac. Depending on the substrate, the ratio of incorporation differed. Also, within each substrate the ratio was different, likely due to variation in the amount of intake.

The flower volatile 2-phenylethanol, originating from L-Phe, was also detected in the nectar but did not, due to known biosynthetic pathways in plants, contribute to the production of MeS. Thus, the ethyl group attached to the benzene ring was not oxidized to benzyl derivatives.

MeS is commonly found to be produced via isochorismic and/or phenylalanine pathways in plants and microorganisms, but the biosynthesis pathway in insects has not been investigated. Our results show that the phenylalanine pathway is an important route for MeS production in butterflies but does not rule out the isochorismic pathway.

By combining known data from the phenyl propanoid pathway in plants and microbes (Fig. 1) with our results revealing the incorporation of labelled aromatic moiety into MeS from a number of intermediate compounds downstream from the pathway of L-Phe to MeS, we suggest that in *P. napi* L-Phe is possibly converted to cinnamic acid by the enzyme similar to phenylalanine lyase (PAL) which further undergoes β-oxidation to benzoic acid, hydroxylation and methylation to MeS. In *P. napi* males, labelled aromatic moiety of L-Phe was also incorporated in benzyl cyanide, suggesting that L-Phe was possibly converted to an aldoxime and further dehydratase produced benzyl cyanide^47,48^. In arthropods, production of phenylacetonitrile from L-Phe via (*E*/*Z*)-phenyl-acetaldoxime is reported for *Chamberlinius hualienensis* Wang (Diplopoda: Paradoxosomatidae)^49^ following the same pathway as described for plants^47,50^. When feeding the adult male butterflies with labelled benzyl cyanide, high incorporation was found in MeS. The pathway of benzyl cyanide conversion to MeS is unknown. The nitrile degradation via nitrilases and nitrilhydratases to acids and ketones is reported in plants, bacteria and fungi^48,51^. The biosynthesis of 2-methoxyphenol is, to our knowledge, not known for insects.

We have shown that adult *P. napi* males utilise L-Phe to produce anti-aphrodisiac pheromone; hence the ability of males to use aromatic precursors for production of MeS could be considered as an advantageous trait which could enable butterflies to relocate L-Phe for other needs, including increase of amino acid content in a nuptial gift. Experimental data revealing the effect of nectar amino acids on the fitness of butterflies vary. In some species, nectar amino acids had no significant positive effect on females’ longevity or fecundity^52–54^, whereas in others adult feeding of amino acids enhanced longevity^55^, increased number of eggs laid^56^, or increased hatching success and produced heavier newly-hatched larvae^57^. In Lepidoptera, spermatophores transferred from males to females at mating are an additional nutritional resource for females^58^. Studies using labelled amino acids acquired during male butterfly larval or adult feeding have demonstrated that those amino acids are built into the spermatophore and transferred to females and subsequently to offspring^58–62^. However, data showing the effect of male acquired nectar amino acids on female reproduction and offspring performance are sparse. Cahenzli and Erhardt^63^ have demonstrated that hatching mass of larvae descended from males fed with amino acid-rich artificial nectar was increased compared to larvae descended from males fed without amino acid nectar mimic. In general, parental-derived essential amino acids were more conserved in the early-instars than non-essential amino acids and are reserved preferentially for protein synthesis^64^. It is of interest to test whether males are able to relocate L-Phe by increasing the amount of this essential amino acid in a nuptial gift after feeding on artificial nectar containing both labelled L-Phe and aromatic precursors of MeS, compared to nectar free of aromatic substances.

In summary, production of the anti-aphrodisiac pheromone MeS by an adult male after feeding on nectar, transfer of the pheromone from male to female during mating, and release of the pheromone by the mated female while displaying the refusal posture was investigated.

The results showed that adult males are able to biosynthesize the anti-aphrodisiac by using either L-Phe or aromatic flower volatiles ingested during feeding on flower nectar. By using stable isotopes we could show that the males are able to restore the level of anti-aphrodisiac titre between matings to at least 40% in compared to the anti-aphrodisiac level the males had after the first mating. Further experiments are needed to identify the remaining steps in the biosynthesis of MeS from L-Phe in male butterflies and whether symbiotic microorganisms can produce the anti-aphrodisiac subsequently used by the butterflies. Moreover, we have shown that in addition to MeS, females received another two compounds: 2-methoxyphenol and benzyl cyanide during mating, wherein MeS was the major compound released during female mate-refusal posture.

## Supplementary information

Supplementary
