# Supplementary material for: Anti-aphrodisiac pheromone, a renewable signal in adult butterflies
Source: Sci Rep. 2019 Oct 3;9:14262. doi: 10.1038/s41598-019-50838-1 (PMC6776535; doi:10.1038/s41598-019-50838-1)
Supplement: Supplementary file 1 — Supplementary [file 41598_2019_50838_MOESM1_ESM.pdf]

Supplementary Information

Anti-aphrodisiac pheromone, a renewable signal in adult butterflies

Raimondas Mozuraitis[1*,2], Rushana Murtazina[3], Javier Zurita[4], Yuxin Pei[5], Leopold Ilag[4,] , Christer Wiklund[1], Anna Karin Borg Karlson[3,6]

1. Department of Zoology, Stockholm University, SE-10691, Stockholm, Sweden
2. Laboratory of Chemical and Behavioural Ecology, Institute of Ecology, Nature Research Centre, Akademijos 2, LT-08412 Vilnius, Lithuania
3. Department of Chemistry, School of Engineering Sciences in Chemistry, Biotechnology and Health, Royal Institute of Technology, KTH, SE-100 44 Stockholm, Sweden
4. Department of Environmental Science and Analytical Chemistry, Stockholm University, SE-10691 Stockholm
5. Northwest A&F University, Department of Applied Chemistry, Yangling, Shaanxi, 712100, People's Republic of China
6. Division of Organic Chemistry, Institute of Technology, Tartu University, Tartu 50411, Estonia

*Corresponding author

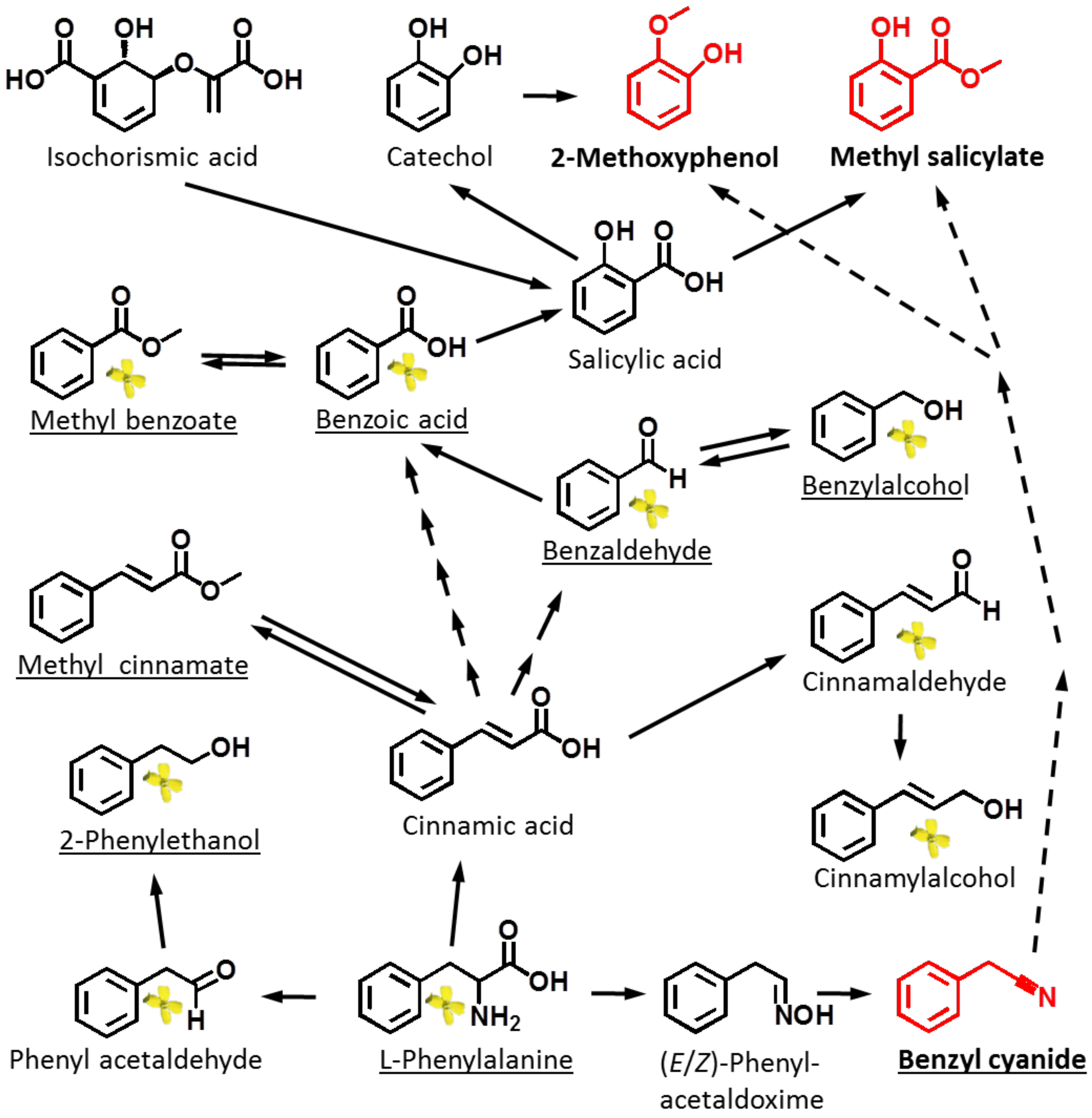

Supplementary Figure 1. B**iochemical pathways leading to the aromatic compounds discussed in the text**.

Structures of compounds shown in red indicate substances including anti-aphrodisiac pheromone methyl salicylate which were exclusively produced by adult males of *Pieris napi* and transferred to the females during mating. Underlined compounds had labelled aromatic moiety and have been fed to the males and transformed to methyl salicylate. Flower sign indicates compounds detected in nectar and/or in floral

scent of *Bunias orientalis* flowers. Solid lines show reported reactions[1-6] and dashed lines indicate incorporations only.

LC-MS analyses of nectar. The nectar sample was collected from Mustard, *Bunias orientalis* L. by using 1µl glass capillary. The synthesis of C_4-NA-NHS was performed according to the method by Yang et al[7]. Standards were prepared individually by weighting the corresponding amounts of nineteen essential amino acids. Derivatization of standard mixture and nectar samples with C_4-NA-NHS was performed and the resulting mixture was then dried under nitrogen flow and reconstituted in 200 µl of 0.3 % acetic acid in water before analysis. HPLC separation was carried out with an Hypersil Gold C18 column (100×2.1 mm, 3 µm particle size; ThermoScientific, USA) and a binary mobile phase (solvent A: 0.3 % acetic acid in water; solvent B: 0.3 % acetic acid in acetonitrile) delivered at a flow rate of 300 µL/min. The linear gradient elution program used was as follows: 0.0 min, 2 % B; 1.0 min, 2 % B; 1.1 min 4% B; 4 min, 4 % B; 15 min, 35 % B; 15.1 min, 90 % B; 20 min, 90 % B; 20.1 min, 2 % B and 25 min, 2 % B. All analyses were performed by injecting 10 µl of either the derivatized sample or the derivatized standard mixture in an Agilent G6410B Triple Quad LC/MS system with electrospray positive ionization and MRM mode. The ion source parameters were optimized as follow: capillary voltage (4,000 V), source temperature (350 °C), gas flow (10 L/min), nebulizer (50 psi), and fragmentor (100 V).

Syntheses of labelled compounds used in the butterfly feeding experiments. All the reactions were run under an atmosphere of dry nitrogen unless otherwise indicated. Anhydrous solvents were transferred using oven-dried syringes. Glassware was oven- or flame dried. Benzene was dried over calcium hydride in a continuous still. Dichloromethane, diethyl ether, THF, and DMF were taken from Meyer's Solvent Dispensing System. All the other solvents and reagents were used as received. ^{1}H-NMR and ^{13}C-NMR spectra were recorded on a Bruker Advance 400 instrument or a Bruker DMX 500 instrument in $CDCl_3$,

using the residual signals from $CHCl_3$ (^1H: δ 7.26 ppm; ^{13}C: δ 77.0 ppm) as internal standard otherwise indicated. Flash chromatography was carried out using SDS silica gel 60 (40–63 µm).

Supplementary Figure 2. **Synthesis of deuterated compounds.**

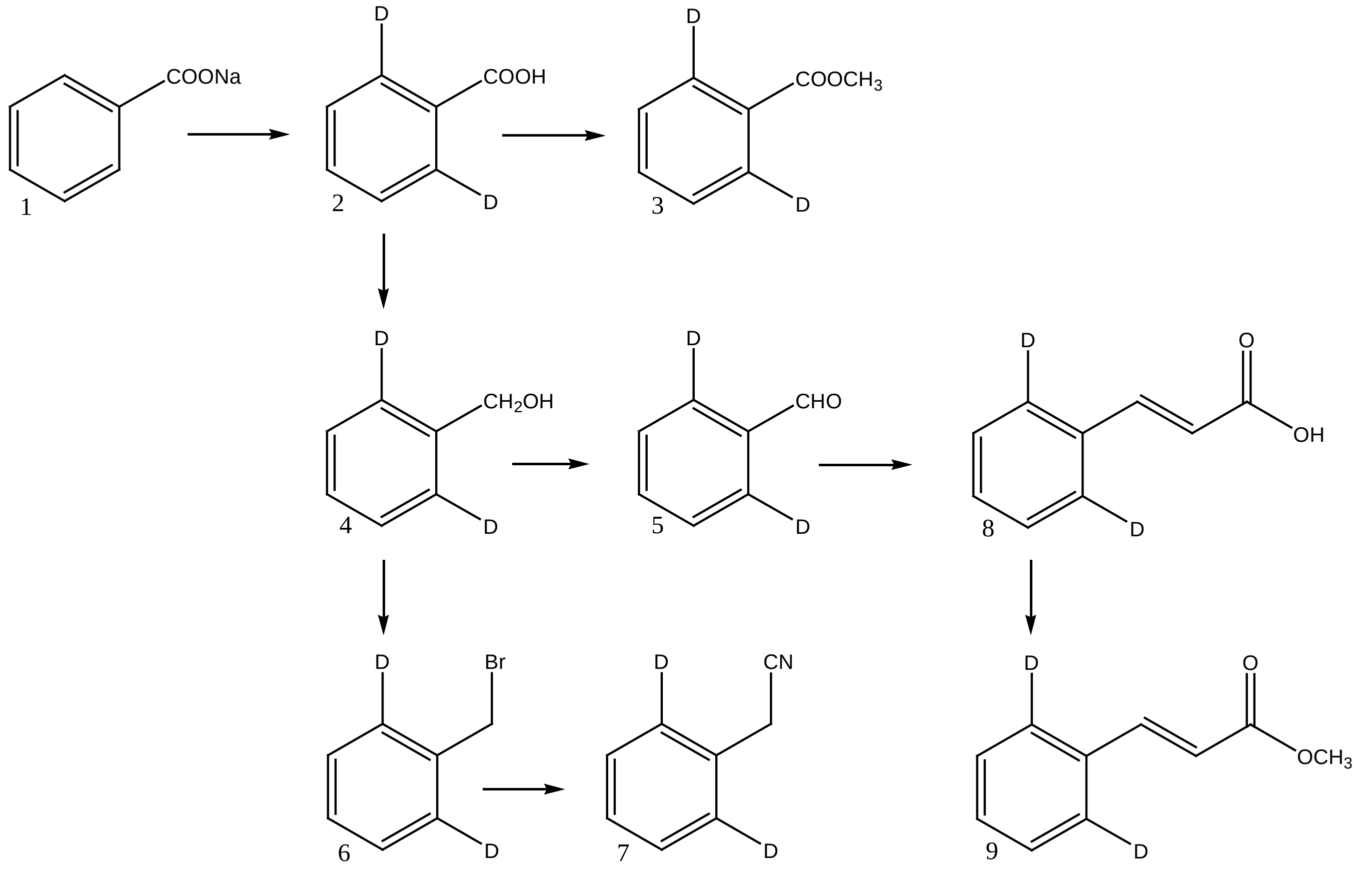

2,6-d$_2$-Benzoic acid (2) was synthesized following the method published by Boyd *et al.*[8] (Supplementary Fig. 2). A mixture of sodium benzoate (0.5 g, 3.47 mmol) and rhodium(III) chloride (0.1 g, 0.48 mmol) dissolved in anhydrous DMF (10 mL) and D_2O (5 mL) was refluxed for 18 h. The reaction mixture was cooled down to rt, acidified with 2M HCl and extracted with Et_2O (3 × 10 mL). The combined extract was washed successively with saturated NaCl solution and water. The organic phase was dried over $MgSO_4$, and the solvent removed under reduced pressure to yield the crude product, which was purified by flash chromatography (2hexane with increasing gra$_d$ient of EtOAc). 2,6-dideuteriobenzoic acid (2; 248

mg, 57%) was obtained as a white crystalline solid. The ^{1}H-NMR and ^{13}C-NMR of the data agreed with those published for the same compound.

Synthesis of 2,6-D_2-methyl benzoate (3). To a solution of 2,6-D_2-benzoic acid (**2**; 248 mg, 2 mmol) in methanol (1 mL, 24.5 mmol) were 3 drops of concentrated sulfuric acid added and the mixture was refluxed under N_2 for 2 h (Supplementary Fig. 2). The reaction mixture was diluted with 1.5 mL water and 3 mL DCM. The aqueous phase was extracted with DCM (5 x 10 mL). The combined organic phase was dried over $MgSO_4$. The solvent was removed under reduced pressure and yellow oil was obtained (**3**; 212 mg, 76%). The ^{1}H-NMR and ^{13}C-NMR of the data agreed with those published for the same compound[8].

2,6-D_2-Benzyl alcohol (4) was synthesized following the method published by Le *et al.*[9] (Supplementary Fig. 2). To a suspension of $LiAlH_4$ (126 mg, 3.3 mmol) in dry Et_2O (6 mL) was added a solution of 2,6-benzoic acid (**2**; 372 mg, 3 mmol) in dry Et_2O (15 mL) at 5 ^{o}C. The reaction mixture was stirred at rt for 15 h, and then quenched at 5 ^{o}C slowly with 0.15 mL water, 0.15 mL of 15% NaOH, and 0.38 mL water. The final mixture was stirred for another 30 min. The precipitate was filtered off and the organic phase of the filtrate was washed with saturated $NaHCO_3$ and brine, dried over $MgSO_4$. The solvent was removed under reduced pressure and compound **4** was obtained as yellow oil (324 mg) quantitatively. The ^{1}H-NMR and ^{13}C-NMR of the data agreed with those published for the same compound[9].

2,6-D_2-Benzaldehyde (5) was synthesized following the method published by Subbarayappa and Patoliya[10] (Supplementary Fig. 2). 324 mg (3 mmol) of **4** in 3.24 mL of dioxane was taken in a 50-mL, round-bottomed flask. N-bromosuccinimide (534 mg, 3 mmol) and 1 drop of water were added. The mixture was stirred at rt for 90 min. The reaction mixture was mixed with 2 mL of 5% sodium thiosulfate solution, and extracted with diethyl ether (5 x 10 mL). The combined organic extract was dried over

Na_2SO_4. Evaporation of the solvent left the crude product, which was purified by flash chromatography (hexane/ethyl acetate, 9:1) to get compound **5** as colorless liquid (51 mg, 0.47 mmol) in 18% yield based on the recovery of starting material compound **4** (53 mg, 0.49 mmol). ^{1}H-NMR (500 MHz, $CDCl_3$) δ ppm 10.03 (s, 1H), 7.64 (t, J = 7.5 Hz, 1H), 7.54 (d, J = 7.5 Hz, 2H).

2,6-D$_2$-Benzyl bromide (6) was synthesized following the method published by Klein *et al.*[11] (Supplementary Fig. 2). Forty eight percent of Hydrobromic acid (395 µL, 3.2 mmol) was mixed with 2,6-D$_2$-benzyl alcohol (**4**; 150 µL, 1.5 mmol). The mixture was refluxed for 4 hrs and then cooled down in ice-water bath. The organic phase was separated with a funnel and washed with cold water. The combined aqueous phase was extracted with DCM. The combined organic phase was dried over $MgSO_4$. The solvent was removed in *vacuo* and red oil obtained which was purified by flash chromatography (hexane/EtOAc, 95:5) affording **6** as colorless oil (158 mg, 61%). ^{1}H NMR (400 MHz, $CDCl_3$) δ ppm 7.35 (d, J = 6.3 Hz, 2H), 7.30 (dd, J = 8.4, 6 Hz, 1H), 4.51 (s, 2H).

2,6-D$_2$-Benzyl cyanide (7) was synthesized following the method published by Soli *et al.*[12] (Supplementary Fig. 2).

Trimethylsilyl cyanide (200 µL, 1.50 mmol) and TBAF (1.5 mL, 1.5 mmol) were added to a stirring solution of 2,6-D$_2$-benzyl bromide (**6**; 140 mg, 0.8 mmol) in acetonitrile (7 mL) under an atmosphere of nitrogen. The reaction mixture was stirred at rt for 15 min. The light yellow reaction mixture was concentrated in *vacuo*, and the resulting syrup was purified by flash chromatography (pentane/CH_2Cl_2, 9:1) to afford 88 mg (93%) of compound **7** as colorless oil. ^{1}H-NMR (500 MHz, $CDCl_3$) δ ppm 7.36 (d, J = 7.4 Hz, 2H), 7.33 (t, J = 7.5 Hz, 1H), 3.75 (s, 2H).

^{13}C-NMR (125 MHz) δ 23.54, 117.88, 127.63 (t), 128.07, 129.05, 129.75 ppm.

Synthesis of 2,6-D_2-cinnamic acid (8). 2,6-D_2-Benzaldehyde (**5,** 804 mg, 7.4 mmol), malonic acid (852 mg, 8.2 mmol), pyridine (0.6 mL, 7,4 mmol) and piperidine (3 mL) were mixed in benzene (70 mL) and heated to reflux for 30 h. NH_4Cl (sat) was added, and the aqueous phase was extracted with Et_2O (Supplementary Fig. 2). The combined organic phases was washed with brine, dried over $MgSO_4$ and evaporated to yield **8** (834 mg, 74%) as slightly yellow crystals.

Synthesis of 2,6-D_2-methyl cinnamate (9). To a solution of 2,6-D_2-cinnamic acid (**8**; 150 mg, 1 mmol) in methanol (1 mL, 24.5 mmol) were 3 drops of concentrated sulfuric acid added and the mixture was refluxed under N_2 for 5 h (Supplementary Fig. 2). The reaction mixture was diluted with 1.5 mL water and 3 mL DCM. The aqueous phase was extracted with DCM (5 x 10 mL). The combined organic phase was dried over $MgSO_4$. The solvent was removed under reduced pressure and whitish solid was obtained (**9**; 131 mg, 80%). ^{1}H-NMR (400 MHz, d_6-DMSO) δ ppm 7.68 (d, 1H, J = 16.5 Hz), 7.43 (s, 3H), 6.65 (d, 1H, J = 16.5 Hz), 3.73 (s, 3H). ^{13}C-NMR (125 MHz, $CDCl_3$) δ 51.68, 117.79, 128.75, 130.26, 134.22, 144.78, 167.41 ppm.

References

1. Maeda, H. & Dudareva, N. The shikimate pathway and aromatic amino acid biosynthesis in plants. *Annu. Rev. Plant Biol.* **63**, 73–405 (2012).
2. Widhalm, J. R. & Dudareva, N. A Familiar ring to it: biosynthesis of plant benzoic acids. *Mol. Plant.* **8**, 83–97 (2015)
3. Irmisch, S., McCormick, A. C., Günther, J., Schmidt, A., Boeckler, G. A., Gershenzon, J., Unsicker, S. B. & Köllner, T. G. Herbivore-induced poplar cytochrome P450 enzymes of the CYP71 family convert aldoximes to nitriles which repel a generalist caterpillar. *The Plant J.* **80**, 1095–1107 (2014).

4. Oinuma, K. I., Hashimoto, Y., Konishi, K, Goda, M., Noguchi, T., Hiroki Higashibata, H. & Kobayashi, M. Novel Aldoxime Dehydratase Involved in Carbon-Nitrogen Triple Bond Synthesis of *Pseudomonas chlororaphis* B23. *J. Biol. Chem.* **278**, 29600–29608, (2003).
5. Beran, F., Köllner, T. G., Gershenzon, J. & Tholl, D. Chemical convergence between plants and insects: biosynthetic origins and functions of common secondary metabolites. *New Phytol.* doi: 10.1111/nph.15718 (2019).
6. Akhtar, T. A. & Pichersky, E. Veratrole Biosynthesis in White Campion. *Plant Physiol*., **162**, 52–62 (2013).
7. Yang, W.C., Mirzaei, H., Liu, X.P. & Regnier, F.E. Enhancement of amino acid detection and quantification by electrospray ionization mass spectrometry. *Anal. Chem*. **78**, 4702–4708 (2006).
8. Boyd, D.R., Sharma, N. D., Harrison, J. S., Malone, J. F., McRoberts, W. C., Hamilton, J. T. G. & Harper, D. B. Enzyme-catalysed synthesis and reactions of benzene oxide/oxepine derivatives of methyl benzoates. *Org. Biomol. Chem.* **6**, 1251–1259 (2008).
9. Le, D. D., Zhang, Y, Chien, D. H. & Moravek, J. Syntheses of N-t-butyl-α-phenylnitrone- α-^{14}C and α-(4-pyridyl-1-oxide)-N-t-butylnitrone- α-^{14}C. *J. Label. Compd. Radiopharm.* **43**, 1119**–**1125 (2000).
10. Subbarayappa, A & Patoliya, P. U. N-Bromosuccinimide: A Facile Reagent for the Oxidation of Benzylic Alcohols to Aldehydes. *Synth. Commun.* **37**, 1571–1577 (2007).
11. Klein, S. M., Zhang, C. & Jiang, Y. L. Simple synthesis of fresh alkyl iodides using alcohols and hydriodic acid. *Tetrahedron Letters* **49**, 2638–2641 (2008).
12. Soli, E. D., Manoso, A.S., Patterson, M. C., DeShong, P., Favor, D. A., Hirschmann, R. & Smith A. B. Azide and Cyanide Displacements via Hypervalent Silicate Intermediates.; *J. Org. Chem*. **64**, 3171–3177 (1999).
